# Comparison of echocardiographic indices of right ventricular systolic function and ejection fraction obtained with continuous thermodilution in critically ill patients

**DOI:** 10.1186/s13054-019-2582-7

**Published:** 2019-09-13

**Authors:** Romain Barthélémy, Xavier Roy, Tujia Javanainen, Alexandre Mebazaa, Benjamin Glenn Chousterman

**Affiliations:** 1 Department of Anaesthesia and Critical Care, Lariboisière Hospital, DMU Parabol, APHP.Nord, Paris, France; 2 Inserm UMR-S942, Mascot, Paris, France; 3 Université de Paris, Paris, France; 4 Réanimation Chirurgical Polyvalente, Hôpital Lariboisière, 2 rue Ambroise Paré, 75475 Paris Cedex 10, France

**Keywords:** Critical illness, Ventricular dysfunction, Right, Catheterization, Swan-Ganz, Thermodilution, Echocardiography, 2D, Echocardiography, Doppler

## Abstract

**Background:**

Though echocardiographic evaluation assesses the right ventricular systolic function, which of the existing parameters best reflects the right ventricular ejection fraction (RVEF) in the critically ill patients is still uncertain. We aimed to determine the relationship between echocardiographic indices of right ventricular systolic function and RVEF.

**Methods:**

Prospective observational study was conducted in a mixed Surgical Intensive Care Unit (Hôpital Lariboisière, Paris, France) from November 2017 to November 2018. All critically ill patients monitored with a pulmonary artery catheter were assessed. We collected echocardiographic indices of right ventricular function (tricuspid annular plane systolic excursion, TAPSE; peak systolic velocity of pulsed tissue Doppler at lateral tricuspid annulus, S′; fractional area change, FAC; right ventricular index of myocardial performance, RIMP; isovolumic acceleration, IVA; end-diastolic diameter ratio, EDDr) and compared them with the RVEF obtained from continuous volumetric pulmonary artery catheter.

**Results:**

Twenty-five patients were analyzed. Admission diagnosis was acute heart failure in 11 patients and septic shock in 14 patients. Median age was 70 years [57–80], norepinephrine median dose was 0.29 μg/kg/min [0.14–0.50], median Sequential Organ Failure Assessment score was 12 [10–14], and mortality at day 28 was 56%. When compared to RVEF, TAPSE had the highest correlation coefficient (rho = 0.78, 95% CI 0.52 to 0.89, *p* < 0.001). S′ was also correlated to RVEF (rho = 0.64, 95% CI 0.60 to 0.80, *p* = 0.001) whereas FAC, RIMP, IVA, and EDDr did not. TAPSE lower than 16 mm, S′ lower than 11 cm/s, and EDDr higher than 1 were always associated with a reduced RVEF.

**Conclusions:**

We found that amongst indices of right ventricular systolic function, TAPSE and S′ were well correlated with thermodilution-derived RVEF in critically ill patients.

**Electronic supplementary material:**

The online version of this article (10.1186/s13054-019-2582-7) contains supplementary material, which is available to authorized users.

## Background

Right ventricular (RV) dysfunction is frequently encountered in intensive care unit (ICU) and has been associated with poor outcome in many acute clinical situations such as respiratory failure, septic shock, acute heart failure, pulmonary embolism, myocardial infarction, and acute decompensated pulmonary hypertension [1–3]. It occurs when RV preload and/or afterload are increased and may lead to acute right ventricular failure with systemic congestion and/or low cardiac output. However, this syndrome has no consensual definition, and its diagnosis is mostly based on a complex association of context, clinical presentation, and echocardiographic evaluation [1, 3]. The gold standard for measurement of RV systolic function is considered to be RV ejection fraction (RVEF) measured by cardiac MRI [2], but the use of this technique is impractical in hemodynamically impaired critically ill patients requiring mechanical ventilation and continuous infusion of inotropes or vasopressors.

According to international consensus, transthoracic echocardiography (TTE) is the first-line recommended exam when investigating the hemodynamic failure of ICU patients [4]. International societies of echocardiography recommend to assess RV systolic function with several indices [5]. Some of the proposed indices have been compared with cardiac MRI [6–8]. However, none have been validated with the RVEF in ICU patients [9], and which of those parameters best reflects RVEF in the critically ill is uncertain.

To investigate this question, we compared echocardiographic indices of RV systolic function to RVEF measured by continuous volumetric pulmonary artery catheter (PAC). This method is not the gold standard for RVEF measurement and was chosen as the best bedside method available that is not based on echocardiography.

## Material and methods

This is a prospective observational study aiming to compare TTE indices of RV systolic function to RVEF measured by continuous volumetric PAC. This study was approved by an institutional ethics committee (*Comité d’éthique de la Société Française d’Anesthésie-Réanimation*, IRB 00010254-2016-034) which waived the need of signed informed consent.

### Patients

The study was conducted between November 2017 and November 2018 in a 20-bed mixed surgical ICU without post-cardiac surgery patients (Hôpital Lariboisière, Paris, France). Our ICU has a long experience of PAC use. In our ICU protocol, PAC is indicated as according to international consensus, i.e., in case of refractory shock not responding to initial therapy and suspicion of RV failure [4]. Postoperative cardiac surgery or scheduled PAC monitoring is not referred to our ICU.

Inclusion criteria were all consecutive patients in whom a PAC had been used for hemodynamic monitoring for less than 24 h.

Exclusion criteria were age < 18 years old, insufficient TTE quality to allow the measurements of indices of RV systolic function (i.e., inability of the investigator to obtain a regular apical 4-chamber view to allow accurate measurements of ventricle dimensions, and accurate time-motion or Doppler measurements without angle correction), cardiac dysrhythmias responsible of irregular echocardiographic patterns, and parameters known to interfere with continuous volumetric PAC performance: hypothermia (central temperature < 35 °C), intracardiac shunt, and tricuspid regurgitation (TR) evaluated after PAC insertion classified as severe according to recommendations [10] (Additional file 1).

### Data collection

Patients’ demographic characteristics, diagnosis at admission, hemodynamic status (heart rate, mean arterial pressure, systolic arterial pressure, diastolic arterial pressure, mixed venous oxygen saturation, arterial lactate), current vasopressor and inotrope treatment, settings of mechanical ventilation, Simplified Acute Physiology Score (SAPS II) at admission, current Sequential Organ Failure Assessment (SOFA), comorbidities, Charlson comorbidity index, and mortality at day 28 were collected.

#### Pulmonary artery catheter

PAC with continuous cardiac output and volumetric measurement (Swan-Ganz CCOmbo V, Edwards Lifesciences, Irvine, CA, USA) was connected to the bedside monitor for pressure measurement (Intellivue MP70, Philips Electronics Nederland B.V., Eindhoven, The Netherlands). Position was checked on the post-insertion chest X-ray or any other more recent X-ray. Zeroing at the phlebostatic level was performed, and waveform of pulmonary artery pressure (PAP) and right atrial pressure (RAP) checked to ensure good positioning. Quality of the arterial pressure signal was assessed with a fast-flush test ensuring no abnormal signal damping. Criteria for the adequate wedge position of the tip of the catheter were as follows: (1) change of the pulmonary artery waveform to atrial waveform during occlusion, (2) a mean end-expiratory pulmonary capillary wedge pressure (PCWP) lower than the diastolic pulmonary arterial pressure (dPAP), and in case of mechanical ventilation, (3) a ratio of induced variations of PCWP to induced variations of systolic pulmonary arterial pressure (sPAP) lower than 1.5 [11, 12].

Continuous volumetric PAC was also connected to the Vigilance II monitor (Edwards Lifesciences, Irvine, CA, USA) for continuous cardiac output monitoring and computation of the RVEF. Briefly, the continuous PAC is supplied with a heating filament located in the right ventricle and a distal thermistor located in the pulmonary artery. The above criteria ensure the correct position of the catheter. A pseudorandom binary heating pattern delivered to the filament and the temperature variation registered by the thermistor are combined by a cross-correlation algorithm to compute the thermodilution curve every 54 s [13, 14]. The largest reported time delay of the device after therapeutic intervention is lower than 20 min; thus, a 20-min stable period without intervention was required before hemodynamic evaluation [14]. During this period, doses of medications and mechanical ventilation settings were not modified, and fluid boluses were avoided. The cardiac output (CO) is determined from the Stewart and Hamilton principle [15, 16] adapted to thermodilution by Fegler [17, 18]. The stroke volume (SV) is computed from the CO divided by the heart rate obtained from the ECG of the bedside monitor. The ratio of end-systolic volume to end-diastolic volume is derived from the wash-out portion of the thermodilution curve and the heart rate; RVEF can therefore be calculated [19, 20].

To ensure concomitant evaluation of both echocardiographic and PAC indices, the following parameters were collected just before the TTE was performed: systolic pulmonary arterial pressure (sPAP), diastolic pulmonary arterial pressure (dPAP), mean pulmonary arterial pressure (mPAP), pulmonary capillary wedge pressure (PCWP), right atrial pressure (RAP), cardiac output (CO), stroke volume (SV), right ventricle end-diastolic volume (RVEDV), right ventricle end-systolic volume (RVESV), and RVEF. Coronary perfusion pressure (CPP) was secondarily calculated as DAP minus RAP.

#### Transthoracic echocardiography

Echocardiographic data were obtained from a 1.5–3.6-MHz cardiac probe connected to a Vivid i echograph (GE Healthcare, Wauwatosa, WI, USA). To ensure consistency in the measurements of the echocardiographic indices, all TTE were performed by a limited number of physicians with advanced expertise in critical care echocardiography (R.B., X.R., or T.J.). Before the beginning of the inclusions, they all studied the latest recommendations of the American Society of Echocardiography endorsed by the European Association of Echocardiography [5] and performed several exams all together to harmonize their practices. ETT parameters were measured and calculated off-line on acquired images, blinded from the PAC values, on an average of 3 measurements.

The following RV systolic function parameters were measured as recommended by international guidelines [5]: fractional area change (FAC), tricuspid annular plane systolic excursion (TAPSE) in M-mode, and indices derived from the pulsed tissue Doppler at lateral tricuspid annulus—peak systolic velocity (S′), RV index of myocardial performance (RIMP), and isovolumic acceleration (IVA). RIMP is defined as the ratio of isovolumic time and ejection time; isovolumic time is calculated as the tricuspid opening time minus ejection time. Time interval is measured from a single beat. IVA is defined as the ratio of peak isovolumic myocardial velocity and time to peak velocity. The onset of myocardial acceleration is at the zero-crossing point of myocardial velocity during isovolumic contraction (Additional file 1).

Other RV parameters not directly related to systolic function were also recorded: RV to left ventricle (LV) end-diastolic diameter ratio (EDDr) and peak TR velocity by continuous waved Doppler [1]. EDDr is defined as the ratio of the basal diameter of RV to LV; basal diameter is consensually defined as the maximal short-axis dimension in the basal one third of the right ventricle seen on the 4-chamber view [5]. sPAP was calculated as the sum of the TR maximal velocity pressure gradient (according to the simplified Bernoulli equation) and the measured RAP [21]. The following LV parameters were also recorded: LV outflow tract diameter, septum and posterior wall thicknesses, LV diastolic diameter, LV ejection fraction (LVEF), mitral inflow Doppler velocity (E and A waves), pulsed waved tissue Doppler velocity at lateral mitral annulus (e′ wave), and velocity-time integral of pulsed waved Doppler at LV outflow tract.

### Statistical analysis

The results are expressed as median [interquartile range] for continuous variables and number (percentage) for categorical variables. Spearman’s correlations and 95% confident intervals (CI) were performed amongst all indices. Missing data were handled by pairwise deletion. Results of correlations are presented in a graphical correlation matrix. Tested variables were ordered using a hierarchical clustering method. A *p* value less than 0.05 was considered as significant.

Specificity (Sp), sensitivity (Se), positive predictive value (PPV), and negative predictive value (NPV) to detect a reduced RVEF were determined for all indices at the recommended thresholds [5]. 95% CI were computed with 2000 bootstrap resampling of the receiver operating characteristics curves. Different thresholds for reduced RVEF are proposed in the literature, usually ranging from 50% (moderately reduced) to 30% (severely reduced) [22]. We chose a cutoff value of 35% as proposed by Vanderpool et al. [23] because it is the threshold that best predicts long-term outcomes. We also tested a lower threshold of 25% to take into account the reported bias of the continuous thermodilution method [24, 25].

All statistical analyses were performed using R statistical software version 3.4.3 (R Core Team, 2017, R Foundation for Statistical Computing, Vienna, Austria, https://www.R-project.org). The visualization of the correlation matrix was drawn with the R package “corrplot” version 0.84 (T. Wei and V. Simko, 2017, https://github.com/taiyun/corrplot). The diagnostic accuracy statistics were performed with the R package “pROC” version 1.1.00 (X. Robin et al., 2011, https://web.expasy.org/pROC/).

## Results

### Patients

Thirty-two consecutive patients met the inclusion criteria during the study period. A diagram depicting the flow of patients is provided in Additional file 2. Two patients could not be included because of the absence of investigators, four patients because of insufficient image quality, and one patient because of a severe tricuspid regurgitation.

Twenty-five patients were analyzed. Admission diagnosis was acute heart failure in 11 patients and septic shock in 14 patients. The median delay between admission and insertion of PAC was 3 [2–4] days.

### Clinical and hemodynamic characteristics

A reduced RVEF was present in 16 patients (64%). At the time of evaluation, 24/25 patients were ventilated, 22/25 received norepinephrine at a median dose of 0.29 μg/kg/min [0.14–0.50], and 4/25 received dobutamine at a median dose of 5 μg/kg/min [5–5]. The median Sequential Organ Failure Assessment score was 12 [10–14]. Mortality at day 28 was 56%. RV free-wall thickness could not be measured for 9 patients, FAC could not be obtained for 2 patients, and EDDr could not be obtained for one patient. Clinical and hemodynamic characteristics of patients are reported respectively in Tables 1 and 2.

**Table 1:** Characteristics of patients at the time of evaluation

Characteristics	Values
Age (years)	70 [57–80]
Female gender	10 (40%)
BMI (kg/m^2^)	24 [21–29]
Admission diagnosis	
Acute heart failure	11 (44%)
Septic shock	14 (66%)
Charlson comorbidity index	6 [2–7]
Comorbidities	
HFrEF	3 (12%)
HFpEF	5 (20%)
COPD	8 (32%)
Pulmonary hypertension	6 (24%)
None	11 (44%)
SAPS II	57 [46–65]
SOFA score	12 [10–14]
Mortality at day 28	14 (56%)
Time from ICU admission (days)	3 [2–4]
Mechanical ventilation	24 (96%)
Tidal volume (mL/kg)	6.5 [6.0–7.1]
Plateau pressure (cmH_2_0)	20 [13–24]
End-expiratory pressure (cmH_2_0)	7 [5–10]
Hemodynamic support	23 (92%)
Norepinephrine	22 (88%)
Norepinephrine dose (μg/kg/min)	0.29 [0.14–0.50]
Dobutamine	4 (16%)
Dobutamine dose (μg/kg/min)	5 [5–5]

Values are median [interquartile range] and number (percentage)

*BMI* body mass index, *HFrEF* heart failure with reduced ejection fraction, *HFpEF* heart failure with preserved ejection fraction, *COPD* chronic obstructive pulmonary disease, *SAPS* simplified acute physiologic score, *SOFA* Sequential Organ Failure Assessment, *ICU* intensive care unit

**Table 2:** Hemodynamics at the time of evaluation

Variables	Values	Missing data, *n* (%)
Systemic hemodynamics		
Heart rate (bpm)	94 [82–105]	
MAP (mmHg)	76 [71–85]	
SAP (mmHg)	103 [98–138]	
DAP (mmHg)	58 [53–67]	
SvO_2_ (%)	71 [63–77]	
Lactate (mmol/L)	2.2 [1.5–3.7]	
PAC pressures		
RAP (mmHg)	9 [6–10]	
sPAP (mmHg)	39 [31–44]	
dPAP (mmHg)	19 [15–25]	
mPAP (mmHg)	25 [21–31]	
PCWP (mmHg)	11 [9–13]	
CPP (mmHg)	51 [47–55]	
PAC thermodilution		
CO (L/min)	4.9 [3.8–6.1]	
SV (mL)	51 [38–77]	
RVEDV (mL)	201 [178–222]	
RVESV (mL)	141 [109–158]	
RVEF (%)	30 [20–37]	
Reduced RVEF	16 (64%)	
Transthoracic echocardiography		
CO (L/min)	4.7 [3.8–6.0]	0
SV (mL)	49 [36–66]	0
LVEF (%)	50 [35–60]	0
FAC (%)	29 [23–40]	2 (8%)
TAPSE (mm)	19 [15–24]	0
S′ (cm/s)	12 [9–15]	0
RIMP	0.81 [0.69–1.03]	0
IVA (m/s^2^)	2.1 [1.5–3.6]	0
EDDr	0.81 [0.74–0.94]	1 (4%)
sPAP (mmHg)	34 [31–41]	7 (28%)
Free wall thickness (mm)	3 [2–4]	9 (36%)

Values are median [interquartile range] and number (percentage). Reduced RVEF is defined as lower than 35% (see the “Material and methods” section)

*MAP* mean arterial pressure, *SAP* systolic arterial pressure, *DAP* diastolic arterial pressure, *SvO*_*2*_ mixed venous oxygen saturation, *vPAC* pulmonary artery catheter, *RAP* right atrial pressure, *sPAP* systolic pulmonary arterial pressure, *dPAP* diastolic pulmonary arterial pressure, *mPAP* mean pulmonary arterial pressure, *PCWP* pulmonary capillary wedge pressure, *CPP* coronary perfusion pressure, *CO* cardiac output, *SV* stroke volume, *RVEDD* right ventricle end-diastolic volume, *RVESV* right ventricle end-systolic volume, *RVEF* right ventricular ejection fraction, *LVEF* left ventricular ejection fraction, *FAC* fractional area change, *TAPSE* tricuspid annular plane systolic excursion, *S′* pic systolic velocity of pulsed tissue Doppler at tricuspid annular, *RIMP* right ventricular index of myocardial performance, *IVA* isovolumic acceleration, *EDDr* end-diastolic diameter ratio

### Correlations between echocardiographic indices of right ventricular systolic function and right ventricular ejection fraction

**Fig. 1:**
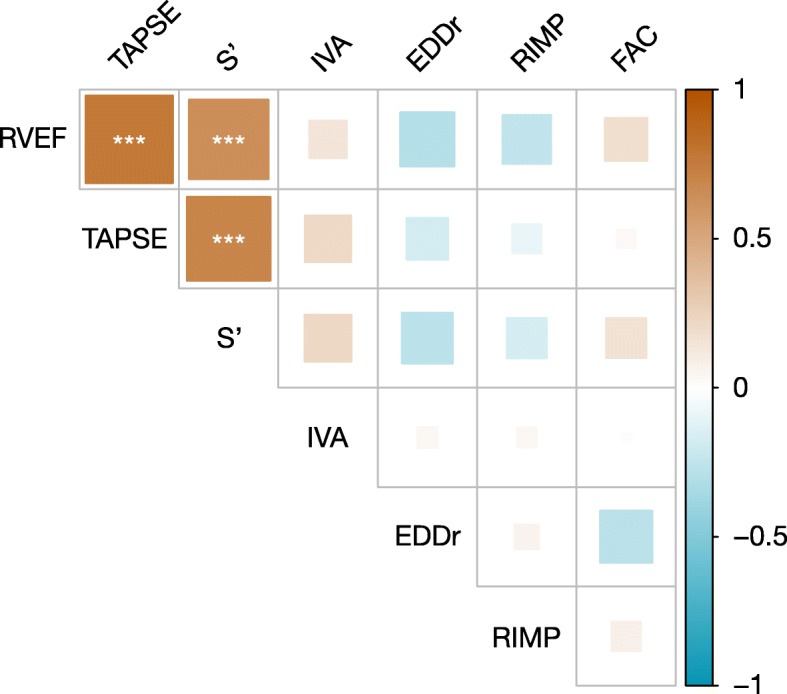
Correlation matrix of transthoracic echocardiography parameters of right ventricular systolic function parameters and right ventricular ejection fraction. RVEF, right ventricular ejection fraction; TAPSE, tricuspid annular plane systolic excursion; S′, peak systolic velocity of pulsed tissue Doppler at tricuspid annulus; IVA, isovolumic acceleration; EDDr, end-diastolic diameter ratio; RIMP, right ventricular index of myocardial performance; FAC, fractional area change. Spearman’s correlations are computed amongst all variables. Positive correlations are represented by red squares and negative correlations by blue squares. Larger squares and darker colors represent higher correlation coefficient. ***p < 0.001, **p < 0.01, *p < 0.05

**Fig. 2:**
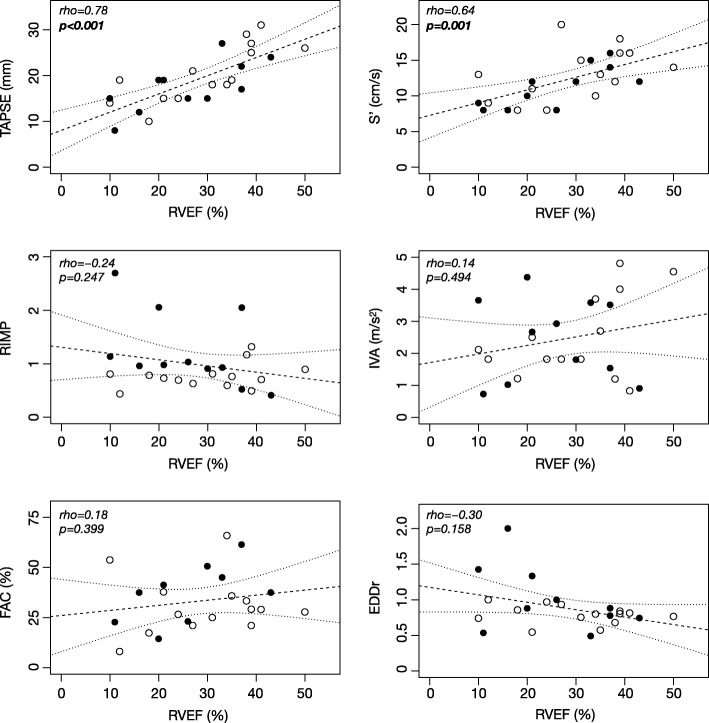
Correlations between right ventricular ejection fraction and the main echocardiographic parameters of right ventricular function. RVEF, right ventricular ejection fraction; TAPSE, tricuspid annular plane systolic excursion; S′, peak systolic velocity of pulsed tissue Doppler at tricuspid annulus; RIMP, right ventricular index of myocardial performance; IVA, isovolumic acceleration; EDDr, end-diastolic diameter ratio; RIMP, right ventricular index of myocardial performance; FAC, fractional area change. Symbol represents admission category: solid circles (●) are for acute heart failure and hollow circles (○) for septic shock. Dashed lines represent the regression lines and dotted lines the 95% confident interval of the regression lines. Rho and *p* value are for Spearman’s correlation

Results of Spearman’s correlations between TTE and continuous volumetric PAC parameters of RV systolic function are shown in Figs. 1 and 2. Correlation matrix of all RV echocardiographic indices and PAC variables is presented in the supplementary material (Additional file 3).

When compared to RVEF, TAPSE had the highest correlation coefficient (rho = 0.78, 95% CI 0.52 to 0.89, *p* < 0.001). S′ was also correlated to RVEF (rho = 0.64, 95% CI 0.60 to 0.80, *p* = 0.001) whereas FAC, RIMP, IVA, and EDDr did not (respectively rho = 0.18, 95% CI − 0.24 to 0.56, *p* = 0.399; rho = − 0.24, 95% CI − 0.58 to 0.17, *p* = 0.247; rho = 0.14, 95% CI–0.17 to 0.58, *p* = 0.494; rho = − 0.30, 95% CI − 0.68 to 0.03, *p* = 0.158).

When evaluating correlations amongst indices, we only found TAPSE and S′ to be significantly correlated (rho = 0.71, 95% CI 0.37 to 0.84, *p* < 0.001).

### Diagnostic accuracy of reduced right ventricular ejection fraction

The results are presented in Table 3. TAPSE lower than 16 mm, S′ lower than 11 cm/s, and EDDr higher than 1 were always associated with a reduced RVEF. The receiver operating characteristic curves of all indices of reduced RVEF are shown in the supplementary material (Additional file 4). The results with the lower threshold of RVEF (< 25%) are presented in the supplementary material (Additional file 5).

**Table 3:** Diagnostic accuracy of reduced right ventricular ejection fraction with echocardiographic indices at their recommended thresholds

Indices	Sp	Se	NPV	PPV
TAPSE < 16 mm	100 [100–100]	56 [31–81]	56 [45–75]	100 [100–100]
S′ < 10 cm/s	100 [100–100]	38 [63–88]	60 [47–82]	100 [100–100]
RIMP < 0.55	33 [11–67]	94 [81–100]	75 [29–100]	71 [63–84]
IVA < 2.2 m/s^2^	56 [22–89]	56 [31–81]	42 [20–64]	69 [50–91]
FAC < 35%	38 [0–75]	53 [27–80]	30 [0–55]	62 [42–80]
EDDr > 1	100 [100–100]	33 [13–60]	47 [41–60]	100 [100–100]

Reduced RVEF is defined as lower than 35%. The tested threshold of the echocardiographic indices are those recommended in the guidelines [5]

*RVEF* right ventricular ejection fraction, *TAPSE* tricuspid annular plane systolic excursion, *S*′ pic systolic velocity of pulsed tissue Doppler at tricuspid annular, *RIMP* right ventricular index of myocardial performance, *IVA* isovolumic acceleration, *FAC* fractional area change, *EDDr* end-diastolic diameter ratio, *Sp* specificity, *Se* sensitivity, *NPV* negative predictive value, *PPV* positive predictive value

## Discussion

In this monocenter study with a limited number of patients, we found that amongst indices of right ventricular systolic function only TAPSE and S′ were well correlated with and could therefore represent good surrogates of thermodilution-derived RVEF in severe critically ill patients.

The recommended method to evaluate RV systolic function is the RVEF measured with cardiac MRI [2]; however, cardiac MRI is difficult to perform in critically ill patients with ventilatory and hemodynamic supports. None of the indices of RV systolic function has been validated against RVEF in critically ill patients [9]. To investigate the relationship between these indices and RVEF in severe patients, we chose to use the RVEF derived from the washout portion of the thermodilution curve of a continuous volumetric PAC as reference method [19]. This is a limitation because it is not the gold standard for RVEF, and its accuracy has been debated [26]. It has been shown not to be interchangeable with cardiac MRI in some study of cardiology patients [27, 28] but to have good correlation in another [29]. In ICU patients, our population of interest, it has been historically validated against radio-nuclear angiography [30–33] and considered accurate to monitor patients with septic shock [34]. In the most recent studies in critically ill patients, when compared to three-dimensional transesophageal echocardiography, the reported bias was considered as clinically acceptable [24, 35]. Some studies [24, 28, 29, 35] report a systematic underestimation of the RVEF with thermodilution, a well-known technical limitation [25]. However, this limitation is unlikely to significantly impact our main results because we investigated echocardiographic indices as continuous physiological variables and their correlations with RVEF rather than predictors of true values. In addition, assuming that PAC may underestimate RVEF, we tested a lower cutoff value for the definition of reduced RVEF. It did not significantly modify our results. Another limitation could be the presence of TR. Severe TR was an exclusion criterion, but mild or moderate TR was present in a significant number of patients in our study. However, the continuous thermodilution has less limits than the cold bolus method, especially regarding dysrhythmias and TR [36, 37].

One strength of our study is that the risk of error with any of the compared method is minimized since our team has a long experience of PAC use and is well aware of its pitfalls and limitations, and TTEs were only performed by a limited number of trained experts with homogenous practices. Good intra- and interobserver variabilities are reported when measuring parameters of RV systolic function [38]. However, we did not specifically evaluate these variabilities in our study, which is a limitation.

Echocardiographic indices are usually proposed as a binary approach of RV dysfunction [8, 9]. Our study shows that TAPSE and S′, because of their good correlation with RVEF, could be reasonably used as continuous surrogates of RVEF and not only as a dichotomized marker of dysfunction. Thus, our results suggest that TAPSE and S′ could therefore be valuable indicators for monitoring the evolution of the RVEF in ICU patients, but it has to be confirmed by a trending study. In addition, they both provide a valuable information when their values are lower than the recommended thresholds (TAPSE < 16 mm or S′ < 10 cm/s) because it can confirm a clinical suspicion of RV dysfunction with a high PPV. However, a value above these cutoffs could not exclude an abnormal RVEF. TAPSE and S′ are also well correlated with each other, which is consistent with previous literature [39]. The close relationship between these two parameters is not surprising since both are obtained from the displacement of the lateral part of the tricuspid annular plane. TAPSE and S′ are thought to be indices of RV longitudinal contraction only, missing free wall abnormalities commonly encountered in acute pulmonary hypertension [40]. In our study, this limitation does not seem to have had a significant impact on the correlation with RVEF. However, due to technical limitation of the echocardiograph we used, we could not measure RV regional dysfunction with free-wall speckle-tracking strain in our study. This could be the subject of a further study.

FAC is known to be well correlated with RVEF in a recent meta-analysis of cardiology studies [6]. In a recent study comparing MRI-based RVEF to echocardiographic indices in non-critically ill patients, FAC appeared to correlate with RVEF better than TAPSE [7]. FAC has the theoretical advantage to be a global index of RV function, incorporating both longitudinal and free-wall contractility [9]. Thus, FAC or speckle-tracking strain may give additional information to TAPSE in clinical situations with acute pulmonary hypertension with free-wall abnormalities [8, 41, 42]. However, the feasibility of FAC has been reported around 60% in ventilated critically ill patients, mainly limited by insufficient acoustic window [39], and its interobserver agreement has been questioned [40]. In our study, despite exclusion of 4 patients because of insufficient acoustic window, the investigators were unable to measure the free wall thickness in a substantial number of patients and finally decided not to measure FAC in two patients. This technical limitation might be responsible for insufficient accuracy in the measurement of FAC in the setting of ventilated critically ill patients and could participate in the poor performance of FAC in our study.

RIMP and IVA are thought to be relatively independent of loading conditions, and RIMP is known to encompass both systolic and diastolic components [1, 5]. It is not the case of RVEF which depends on a complex and nonlinear relationship with preload and afterload [23, 43]. This could explain the absence of correlation with RVEF. However, RIMP and IVA do not correlate amongst them nor with any of the other measured parameters of RV function. What information is brought to physicians by these two indices in the critically ill patient remains to be explored.

EDDr was not correlated to RVEF as a continuous variable, but all patients with RV dilation on the TTE (EDDr > 1) had a reduced RVEF. However, a decrease in RVEF can be missed by a simple measure of RV dilation. This is expected as dilation of the RV is one of the determinants of reduced RVEF, but not the only one.

All other tested indices were neither correlated to RVEF nor efficient classifiers of reduced RVEF at the tested cutoffs. Thus, our study shows that amongst all the recommended indices of RV systolic function, TAPSE and S′ are those who have the most determinants in common with RVEF. However, our results do not imply that other indices of RV function should not be used since they bring additional information to RVEF that might help the clinician when assessing RV systolic function.

The present study focuses on severe critically ill patients with a high mortality rate, high SAPS II and SOFA scores, elevated lactate level, and who required both vasopressors and mechanical ventilation while most of the available literature regarding validation of echocardiographic indices of RV systolic function with RVEF were performed on stable cardiology patients. The investigation of RV function in patients with refractory shock is clinically relevant because it is a subset of patient with poor outcome in which clinician should evoke the possibility of RV failure in the hemodynamic picture because it can modify the management of the supportive therapies (fluid balance, choice of vasoactive drugs, settings of mechanical ventilation) and potentially impact outcome [1–3, 44]. In our cohort, a substantial number of patients had a low RVEF that could have been missed if it has not been investigated.

Our study has some limitations. It is a monocenter study with a limited number of patients. Thus, it is likely that we only identified the strongest correlations and cannot exclude that a larger study may reveal significant correlations between the other indices. We compared echocardiographic indices of RV systolic function to RVEF in patients with primary septic shock or acute heart failure in which poor response to initial therapy leads to the decision of PAC monitoring by the attending physician. These are already resuscitated patients who achieved standard hemodynamic targets but remain dependent on vasopressor and/or inotropes and do not recover from their organ failures or have persistent hyperlactatemia. None of our patients had myocardial infarction, and CPP did not appear to be limiting. It is a particular severe subtype of patients and represents a selection bias that prevents generalization of our results to all critically ill patients. These are preliminary data that advocate for a larger study to confirm our results. Another limitation is that these indices were not tested in patients with non-resuscitated shock or primary RV failure at ICU admission (pulmonary embolism with shock or RV infarction with shock). Our results should not be extrapolated to these clinical scenarios.

## Conclusion

We found that amongst indices of right ventricular systolic function, TAPSE and S′ were well correlated with thermodilution-derived RVEF in critically ill patients.

## Additional files

Additional file 1: Criteria for severe tricuspid regurgitation and Figure of Doppler tissue velocities and time intervals obtained at lateral tricuspid valve annulus (DOCX 218 kb)

Additional file 2: Flow chart of the study (PDF 14 kb)

Additional file 3: Correlation matrix of continuous volumetric pulmonary arterial catheter and echocardiographic parameters of right ventricular function (DOCX 596 kb)

Additional file 4: Receiver operating characteristic curves of echocardiographic indices for diagnosis of reduced right ventricular ejection fraction (DOCX 160 kb)

Additional file 5: Diagnostic accuracy of reduced right ventricular ejection fraction (< 25%) with echocardiographic indices (DOCX 17 kb)

## Data Availability

The datasets used and/or analyzed during the current study are available from the corresponding author on reasonable request.

## Abbreviations

CI: Confident interval
CO: Cardiac output
dPAP: Diastolic pulmonary arterial pressure
EDDr: End-diastolic diameter ratio
ICU: Intensive care medicine
IVA: Isovolumic acceleration
LV: Left ventricle
LVEF: Left ventricular ejection fraction
mPAP: Mean pulmonary arterial pressure
MRI: Magnetic resonance imaging
NPV: Negative predictive value
PAC: Pulmonary artery catheter
PAP: Pulmonary arterial pressure
PCWP: Pulmonary capillary wedge pressure
PPV: Positive predictive value
RAP: Right atrial pressure
RIMP: Right ventricular index of myocardial performance
RV: Right ventricle
RVEDV: Right ventricular end-diastolic volume
RVEF: Right ventricular ejection fraction
RVESV: Right ventricular end-systolic volume
SAPS: Simplified acute physiological score
Se: Sensitivity
SOFA: Sequential Organ Failure Assessment
Sp: Specificity
sPAP: Systolic pulmonary arterial pressure
SV: Stroke volume
TAPSE: Tricuspid annular plane systolic excursion
TR: Tricuspid regurgitation
TTE: Transthoracic echocardiography
