# Supplementary material for: Comparison of echocardiographic indices of right ventricular systolic function and ejection fraction obtained with continuous thermodilution in critically ill patients
Source: Crit Care. 2019 Sep 13;23:312. doi: 10.1186/s13054-019-2582-7 (PMC6743193; doi:10.1186/s13054-019-2582-7)
Supplement: Supplementary file 1 — Criteria for severe tricuspid regurgitation and Figure of Doppler tissue velocities and time intervals obtained at lateral tricuspid valve annulus (DOCX 218 kb) [file 13054_2019_2582_MOESM1_ESM.docx]

# Criteria for severe tricuspid regurgitation (TR)

These criteria are based on: Lancellotti P, Tribouilloy C, Hagendorff A, Popescu BA, Edvardsen T, Pierard LA, et al. Recommendations for the echocardiographic assessment of native valvular regurgitation: an executive summary from the European Association of Cardiovascular Imaging. Eur Heart J Cardiovasc Imaging. 2013;14:611–44.

Quantitative:

- Abnormal/flail/large coaptation defect of the tricuspid valve
- Very large central TR jet or eccentric wall-impinging TR jet in color flow (at Nyquist limit of 50–60 cm/s)
- Dense/triangular TR with early peaking in continuous wave signal

Semi-quantitative:

- Vena contracta width > 7 mm (at Nyquist limit of 50–60 cm/s)
- PISA radius > 9 mm with baseline Nyquist limit shift of 28 cm/s
- Systolic flow reversal in hepatic veins
- Dominant E-wave ≥ 1 m/s in the absence of other cause of increased right atrial pressure

Quantitative:

- Effective regurgitant orifice area ≥ 40 mm^2^

# Doppler tissue velocities and time intervals obtained at lateral tricuspid valve annulus.

Based on: Rudski LG, Lai WW, Afilalo J, Hua L, Handschumacher MD, Chandrasekaran K, et al. Guidelines for the echocardiographic assessment of the right heart in adults: a report from the American Society of Echocardiography endorsed by the European Association of Echocardiography, a registered branch of the European Society of Cardiology, and the Canadian Society of Echocardiography. J Am Soc Echocardiogr. 2010;23:685–713.

A’, Peak velocity during atrial contraction; AT, acceleration time; E’, peak velocity during early diastole; ET, ejection time; IVV, peak myocardial velocity during isovolumic contraction; S’, peak velocity during ejection period of systole, TCO, tricuspid valve closing opening time
