# Supplementary figures and images for: Comparison of echocardiographic indices of right ventricular systolic function and ejection fraction obtained with continuous thermodilution in critically ill patients
Source: Crit Care. 2019 Sep 13;23:312. doi: 10.1186/s13054-019-2582-7 (PMC6743193; doi:10.1186/s13054-019-2582-7)

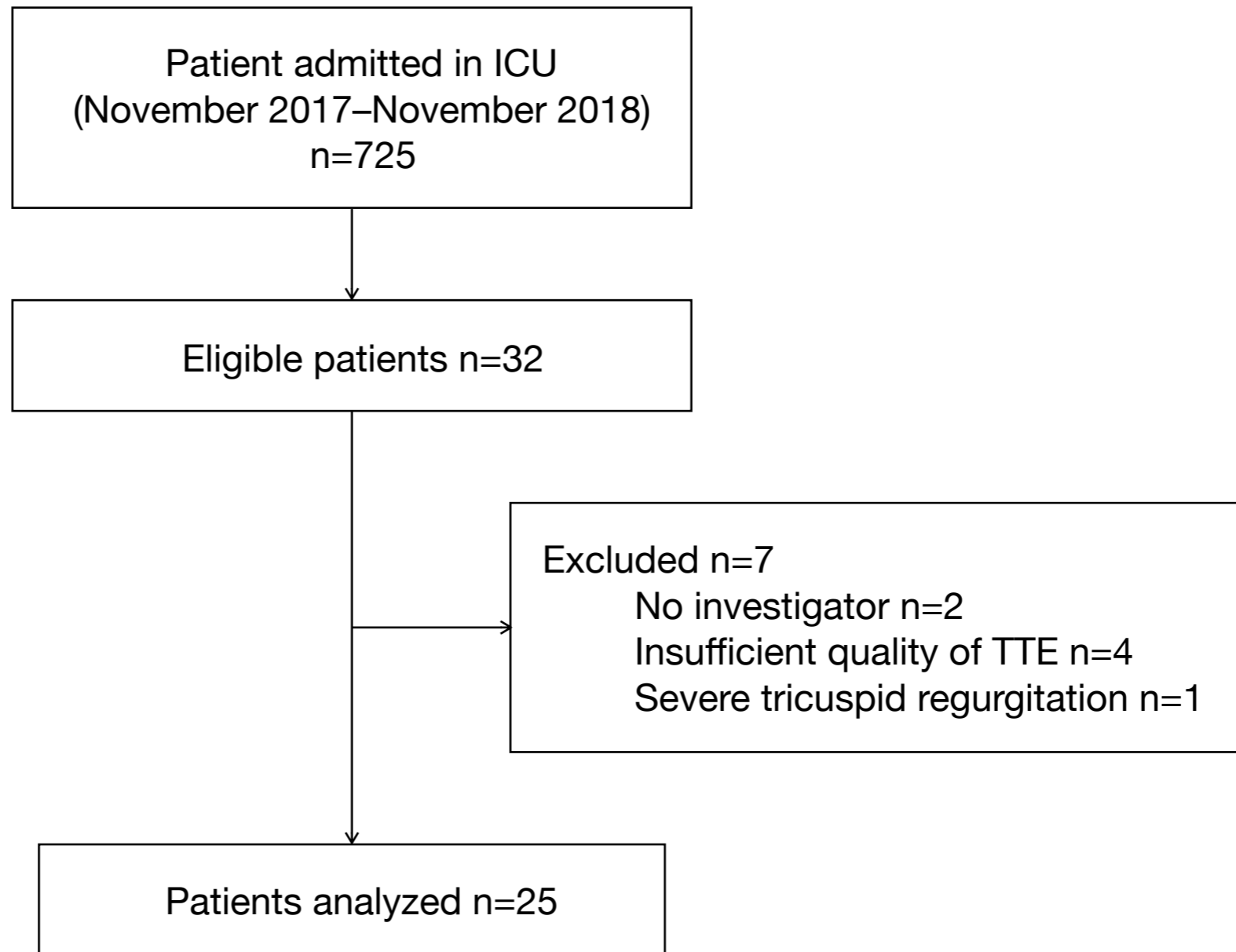

Supplement: Supplementary file 2 — Flow chart of the study (PDF 14 kb) [file 13054_2019_2582_MOESM2_ESM.pdf]
