# Supplementary material for: Comparison of echocardiographic indices of right ventricular systolic function and ejection fraction obtained with continuous thermodilution in critically ill patients
Source: Crit Care. 2019 Sep 13;23:312. doi: 10.1186/s13054-019-2582-7 (PMC6743193; doi:10.1186/s13054-019-2582-7)
Supplement: Supplementary file 3 — Correlation matrix of continuous volumetric pulmonary arterial catheter and echocardiographic parameters of right ventricular function (DOCX 596 kb) [file 13054_2019_2582_MOESM3_ESM.docx]

## Figure S2-A: Correlation matrix of continuous volumetric pulmonary arterial catheter and echocardiographic parameters of right ventricular function

Spearman’s correlations are computed between each variable. Positive correlations are represented by red squares and negative correlations by blue squares. Larger squares and darker colors represent higher correlation coefficient. vPAC: volumetric pulmonary artery catheter, TTE: transthoracic echocardiography, S’: pic systolic velocity of pulsed tissue Doppler at tricuspid annulus, TAPSE: tricuspid annular plane systolic excursion, RVEF: right ventricular ejection fraction, SV: stroke volume, RIMP: right ventricular index of myocardial performance, IVA: isovolumic acceleration, EDDr: end diastolic diameter ratio, mPAP: mean pulmonary arterial pressure, RAP: right atrial pressure, FAC: fractional area change, RVEDV: right ventricular end diastolic volume, RVESV: right ventricular end systolic volume. *** p< 0.001, ** p<0.01, * p<0.05.

## Figure S2-B: Correlation matrix of continuous volumetric pulmonary arterial catheter and echocardiographic parameters of right ventricular function: Spearman’s rho coefficient

Spearman’s correlations are computed between each variable. vPAC: volumetric pulmonary artery catheter, TTE: transthoracic echocardiography, S’: pic systolic velocity of pulsed tissue Doppler at tricuspid annulus, TAPSE: tricuspid annular plane systolic excursion, RVEF: right ventricular ejection fraction, SV: stroke volume, RIMP: right ventricular index of myocardial performance, IVA: isovolumic acceleration, EDDr: end diastolic diameter ratio, mPAP: mean pulmonary arterial pressure, RAP: right atrial pressure, FAC: fractional area change, RVEDV: right ventricular end diastolic volume, RVESV: right ventricular end systolic volume.

## Figure S2-C: Correlation matrix of continuous volumetric pulmonary arterial catheter and echocardiographic parameters of right ventricular function: 95% confident interval of Spearman’s rho coefficient

Spearman’s correlations are computed between each variable. Left: lower 95% confident interval. Right: upper 95% confident interval. vPAC: volumetric pulmonary artery catheter, TTE: transthoracic echocardiography, S’: pic systolic velocity of pulsed tissue Doppler at tricuspid annulus, TAPSE: tricuspid annular plane systolic excursion, RVEF: right ventricular ejection fraction, SV: stroke volume, RIMP: right ventricular index of myocardial performance, IVA: isovolumic acceleration, EDDr: end diastolic diameter ratio, mPAP: mean pulmonary arterial pressure, RAP: right atrial pressure, FAC: fractional area change, RVEDV: right ventricular end diastolic volume, RVESV: right ventricular end systolic volume.

## Figure S2-D: Correlation matrix of continuous volumetric pulmonary arterial catheter and echocardiographic parameters of right ventricular function: p values of Spearman’s rho coefficient

Spearman’s correlations are computed between each variable. vPAC: volumetric pulmonary artery catheter, TTE: transthoracic echocardiography, S’: pic systolic velocity of pulsed tissue Doppler at tricuspid annulus, TAPSE: tricuspid annular plane systolic excursion, RVEF: right ventricular ejection fraction, SV: stroke volume, RIMP: right ventricular index myocardial performance, IVA: isovolumic acceleration, EDDr: end diastolic diameter ratio, mPAP: mean pulmonary arterial pressure, RAP: right atrial pressure, FAC: fractional area change, RVEDV: right ventricular end diastolic volume, RVESV: right ventricular end systolic volume.
