# Supplementary material for: Comparison of echocardiographic indices of right ventricular systolic function and ejection fraction obtained with continuous thermodilution in critically ill patients
Source: Crit Care. 2019 Sep 13;23:312. doi: 10.1186/s13054-019-2582-7 (PMC6743193; doi:10.1186/s13054-019-2582-7)
Supplement: Supplementary file 4 — Receiver operating characteristic curves of echocardiographic indices for diagnosis of reduced right ventricular ejection fraction (DOCX 160 kb) [file 13054_2019_2582_MOESM4_ESM.docx]

## Figure S3-A: Receiver operating characteristic curves of echocardiographic indices for diagnosis of reduced right ventricular ejection fraction (< 35%)

| Indices | AUC | Best Threshold |
| --- | --- | --- |
| TAPSE | 0.90 [0.77–1.00] | 21.5 [16–23] |
| S’ | 0.84 [0.68–1.00] | 11.5 [11–15.5] |
| RIMP | 0.58 [0.31–0.84] | 0.72 [0.42–2.05] |
| IVA | 0.53 [0.25–0.82] | 3.8 [0.8–4.6] |
| FAC | 0.55 [0.30–0.80] | 27 [19–53] |
| EDDr | 0.66 [0.43–0.89] | 0.86 [0.79–0.94] |

TAPSE: tricuspid annular plane systolic excursion, S’: pic systolic velocity of pulsed tissue Doppler at tricuspid annular, RIMP: right ventricular index of myocardial performance, IVA: isovolumic acceleration, FAC: fractional area change, EDDr: end diastolic diameter ratio, AUC: area under the curve. The best thresholds are determined with the Youden index.

## Figure S3-B: Receiver operating characteristic curves of echocardiographic indices for diagnosis of reduced right ventricular ejection fraction (< 25%)

| Indices | AUC | Best Threshold |
| --- | --- | --- |
| TAPSE | 0.87 [0.73–1.00] | 20 [14.5–21.5] |
| S’ | 0.89 [0.76–1.00] | 11.5 [9.5–13.5] |
| RIMP | 0.60 [0.36–0.84] | 0.78 [0.61–2.05] |
| IVA | 0.59 [0.35–0.82] | 2.7 [0.8–4.5] |
| FAC | 0.62 [0.36–0.89] | 23 [18–58] |
| EDDr | 0.68 [0.42–0.94] | 0.86 [0.83–1.17] |

TAPSE: tricuspid annular plane systolic excursion, S’: pic systolic velocity of pulsed tissue Doppler at tricuspid annular, RIMP: right ventricular index of myocardial performance, IVA: isovolumic acceleration, FAC: fractional area change, EDDr: end diastolic diameter ratio, AUC: area under the curve. The best thresholds are determined with the Youden index.
