# Supplementary material for: Comparison of echocardiographic indices of right ventricular systolic function and ejection fraction obtained with continuous thermodilution in critically ill patients
Source: Crit Care. 2019 Sep 13;23:312. doi: 10.1186/s13054-019-2582-7 (PMC6743193; doi:10.1186/s13054-019-2582-7)
Supplement: Supplementary file 5 — Diagnostic accuracy of reduced right ventricular ejection fraction (< 25%) with echocardiographic indices (DOCX 17 kb) [file 13054_2019_2582_MOESM5_ESM.docx]

### Table S1: Diagnostic accuracy of reduced right ventricular ejection fraction (<25 %) with echocardiographic indices

| Indices | Sp | Se | NPV | PPV |
| --- | --- | --- | --- | --- |
| TAPSE < 16 mm | 87 [67–100] | 70 [40–100] | 81 [67–100] | 80 [56–100] |
| S’ < 10 cm/s | 87 [67–100] | 80 [50–100] | 87 [73–100] | 82 [60–100] |
| RIMP < 0.55 | 20 [7–40] | 90 [70–100] | 75 [20–100] | 43 [35–50] |
| IVA < 2.2 m/s^2^ | 53 [27–80] | 60 [30–90] | 67 [45–88] | 46 [27–67] |
| FAC < 35 % | 43 [14–71] | 56 [22–89] | 60 [33–83] | 38 [18–58] |
| EDDr > 1 | 93 [79–100] | 40 [10–70] | 68 [58–82] | 80 [40–100] |

RVEF: right ventricular ejection fraction, FAC: fractional area change, TAPSE: tricuspid annular plane systolic excursion, S’: pic systolic velocity of pulsed tissue Doppler at tricuspid annular, RIMP: right ventricular index of myocardial performance, IVA: isovolumic acceleration, EDDr: end diastolic diameter ratio, Sp: specificity, Se: sensitivity, NPV: negative predictive value, PPV: positive predictive value, Thr.: threshold. Sp, Se, NPV and PPV are computed for the recommended thresholds.
